# Supplementary material for: A ventral striatal learning signal reflecting individual differences in the success of fear extinction
Source: Mol Psychiatry. 2026 Mar 27;31(8):4450–62. doi: 10.1038/s41380-026-03565-9 (PMC13364659; doi:10.1038/s41380-026-03565-9)
Supplement: Supplementary file 1 — Supplementary Information [file 41380_2026_3565_MOESM1_ESM.pdf]

Supplementary Information Elena Andres et al.
Title: A ventral striatal learning signal reflecting individual differences in the success of fear extinction

Andres et al. dataset

Effect	*DFn*	*DFd*	*F*	*p*	*p<.05*	*ges*
Class	1	171	13.31	3.49e-04	*	0.031
Stimulus	1	171	93.80	5.84e-18	*	0.06
Time	1	171	0.09	0.75		0.0001
Class:Stimulus	1	171	0.04	0.83		0.00003
Class:Time	1	171	7.28	0.007	*	0.012
Stimulus:Time	1	171	123.69	5.75e-22	*	0.10
Class:Stimulus:Time	1	171	1.67	0.19		0.001

Supplementary Table 1: Day 1 ANOVA

1) Class fast

Effect	*DFn*	*DFd*	*F*	*p*	*p<.05*	*ges*
Stimulus	1	130	80.84	2.49e-15	*	0.075
Time	1	130	8.83	3.51e-03	*	0.018
Stimulus:Time	1	130	92.87	6.49e-17	*	0.099

2) Class slow

Effect	*DFn*	*DFd*	*F*	*p*	*p<.05*	*ges*
Stimulus	1	41	59.37	1.69e-09	*	0.12
Time	1	41	2.26	0.13		0.01
Stimulus:Time	1	41	65.59	4.85e-10	*	0.21

Supplementary Table 2: Day 1 Within-Class ANOVAs.

Effect	*DFn*	*DFd*	*F*	*p*	*p<.05*	*ges*
Class	1	171	116.91	4.27e-21	*	0.18
Stimulus	1	171	120.39	1.51e-21	*	0.14
Time	1	171	167.07	4.22e-27	*	0.19
Class:Stimulus	1	171	3.95	0.048	*	0.005
Class:Time	1	171	98.64	1.22e-18	*	0.12
Stimulus:Time	1	171	3.95	0.04	*	0.004
Class:Stimulus:Time	1	171	18.47	2.88e-05	*	0.02

Supplementary Table 3: Day 2 ANOVA

1) Class fast

Effect	*DFn*	*DFd*	*F*	*p*	*p<.05*	*ges*
Stimulus	1	130	109.50	5.77e-19	*	0.13
Time	1	130	522.66	2.20e-47	*	0.51
Stimulus:Time	1	130	43.62	9.24e-10	*	0.05

2) Class slow

Effect	*DFn*	*DFd*	*F*	*p*	*p<.05*	*ges*
Stimulus	1	41	31.43	1.56e-06	*	0.22

Time	1	41	3.26	0.07		0.01
Stimulus:Time	1	41	1.45	0.23		0.007

Supplementary Table 4: Day 2 Within-Class ANOVA

Effect	*DFn*	*DFd*	*F*	*p*	*p<.05*	*ges*
Class	1	147	54.58	1.03e-11	*	0.12
Stimulus	1	147	122.00	4.97e-21	*	0.14
Time	1	147	117.43	1.76e-20	*	0.16
Class:Stimulus	1	147	1.76	0.18		0.002
Class:Time	1	147	21.28	8.53e-06	*	0.03
Stimulus:Time	1	147	23.03	3.86e-06	*	0.02
Class:Stimulus:Time	1	147	4.23	0.04	*	0.004

Supplementary Table 5: Day 3 ANOVA

1) Class fast

Effect	*DFn*	*DFd*	*F*	*p*	*p<.05*	*ges*
Stimulus	1	109	88.65	9.22e-16	*	0.15
Time	1	109	261.72	9.61e-31	*	0.35
Stimulus:Time	1	109	45.19	8.49e-10	*	0.07

2) Class slow

Effect	*DFn*	*DFd*	*F*	*p*	*p<.05*	*ges*
Stimulus	1	38	54.51	7.49e-09	*	0.18
Time	1	38	9.57	0.003	*	0.06
Stimulus:Time	1	38	2.50	0.12		0.009

Supplementary Table 6: Day 3 Within-Class ANOVA

Effect	*DFn*	*DFd*	*F*	*p*	*p<.05*	*ges*
Class	1	74	35.52	7.92E-08	*	0.149
Stimulus	1	74	54.33	1.99E-10	*	0.147
Time	1	74	57.08	8.96E-11	*	0.15
Class:Stimulus	1	74	0.19	6.65E-01		0.001
Class:Time	1	74	10.12	2.14E-03	*	0.03
Stimulus:Time	1	74	10.35	1.92E-03	*	0.024
Class:Stimulus:Time	1	74	2.36	1.29E-01		0.005

Supplementary Table 7: Day 3 ANOVA – only placebo

1) Class fast

Effect	*DFn*	*DFd*	*F*	*p*	*p<.05*	*ges*
Stimulus	1	51	36.33	1.86E-07	*	0.164
Time	1	51	111.35	2.00E-14	*	0.312
Stimulus:Time	1	51	17.21	1.27E-04	*	0.063

2) Class slow

Effect	*DFn*	*DFd*	*F*	*p*	*p<.05*	*ges*
Stimulus	1	23	24.93	4.75E-05	*	0.157
Time	1	23	4.99	3.55E-02	*	0.054

Stimulus:Time	1	23	1.13	2.98E-01	0.006

Supplementary Table 8: Day 3 Within-Class ANOVA – only placebo

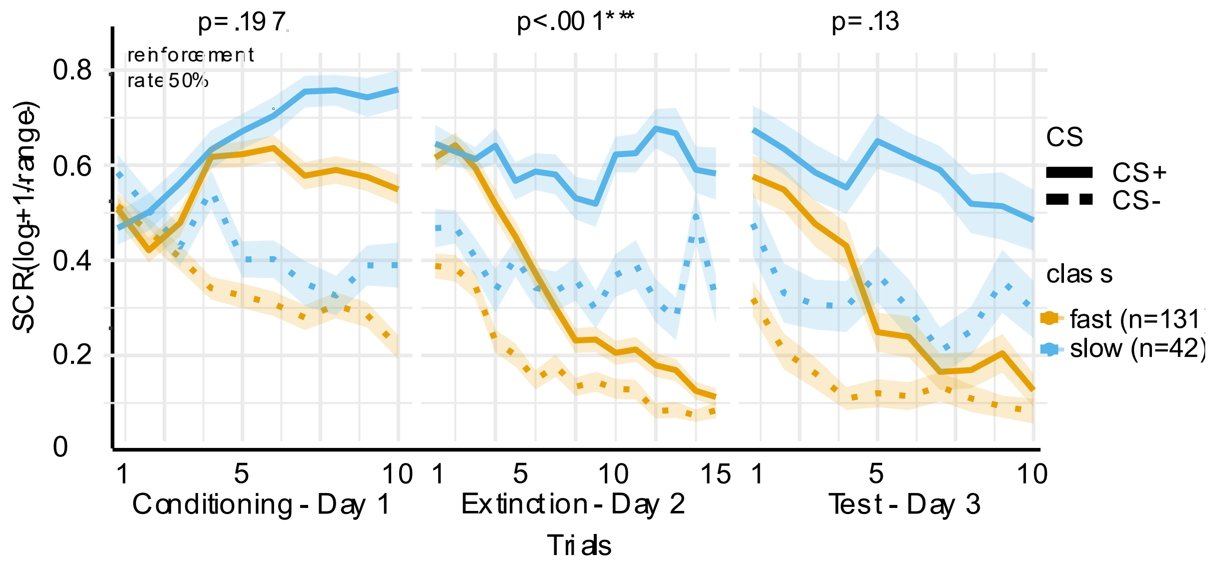

Supplementary Figure 1. Differential SCR dynamics across all phases with only placebo participants on Day 3. Day-3 data contains only the placebo subgroup (n=76) with fast extinguishers (n=52) and slow extinguishers (n=24).

Predictor	*OR*	*2.5%*	*97.5%*
Intercept	0.29	0.02	2.99
STAIT	0.98	0.92	1.04
Dataset(3)	4.01	1.14	18.95
Dataset(2)	0.72	0.17	3.73
Dataset(1)	3.24	0.87	15.77

Predictor	*Estimate*	*Std. Error*	*z value*	*p*
Intercept	–1.23	1.20	–1.03	0.30
STAIT	–0.01	0.03	–0.58	0.55
Dataset(3)	1.38	0.69	1.99	0.046*
Dataset(2)	–0.31	0.76	–0.41	0.67
Dataset(1)	1.17	0.71	1.64	0.10

Supplementary Table 9: Logistic regression results with class membership as the dependent variable and STAI-T and study as predictors.

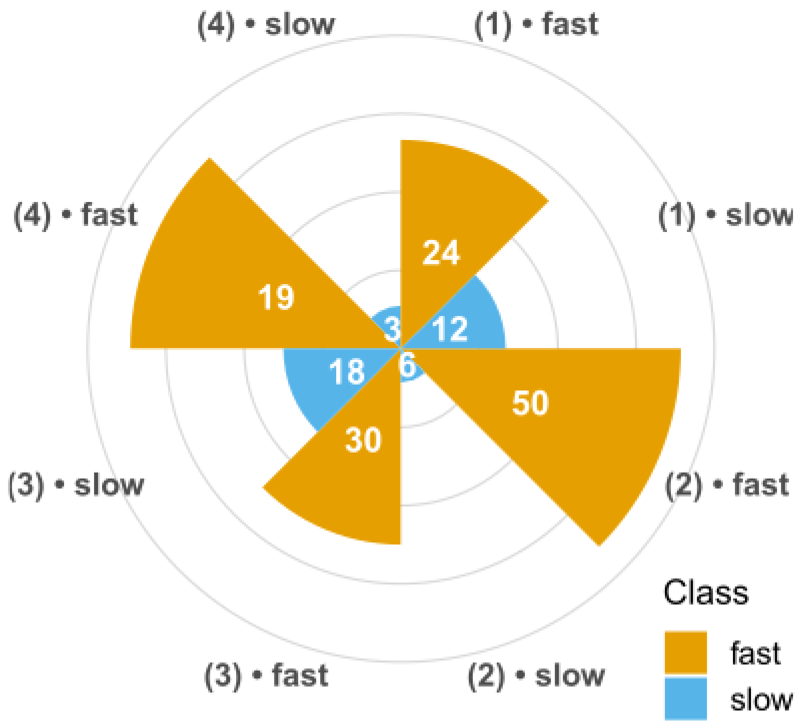

Supplementary Figure 2. Class membership proportions by study. Rings at 0%, 25%, 50%, 75%, 100% (numbers show n). Datasets 1-4 are as described in Methods and Fig. 1.

Effect	*Sum Sq*	*num Df*	*Error SS*	*den Df*	*F value*	*p-value*	*Sig*
(Intercept)	55.74	1	7.06	154	1214.93	< 2.2e-16	***
Class	3.66	1	7.06	154	79.86	1.161e-15	***
Study	0.02	3	7.06	154	0.15	0.92	
Class:Study	0.30	3	7.06	154	2.22	0.08	.
Stimulus	2.55	1	5.35	154	73.62	9.540e-15	***
Class:Stimulus	0.02	1	5.35	154	0.83	0.36	
Study:Stimulus	0.07	3	5.35	154	0.73	0.53	
Class:Study:Stimulus	0.33	3	5.35	154	3.23	0.02	*
Time	3.60	1	5.53	154	100.31	< 2.2e-16	***
Class:Time	2.72	1	5.53	154	75.93	4.352e-15	***
Study:Time	0.18	3	5.53	154	1.71	0.16	
Class:Study:Time	0.15	3	5.53	154	1.42	0.23	
Stimulus:Time	0.25	1	4.34	154	9.16	0.002	**
Class:Stimulus:Time	0.14	1	4.34	154	5.16	0.02	*
Study:Stimulus:Time	0.29	3	4.34	154	3.48	0.01	*
Class:Study:Stimulus:Time	0.03	3	4.34	154	0.43	0.72	

Supplementary Table 10: Follow-up repeated-measures ANOVA on SCR of Day 2 including study to assess potential moderation of class-related effects.

Chalkia et al. dataset

Effect	*DFn*	*DFd*	*F*	*p*	*p<.05*	*ges*
Class	1	163	27.93	3.97e-07	*	0.07
Stimulus	1	163	95.34	5.08e-18	*	0.06
Time	1	163	89.09	3.82e-17	*	0.12
Class:Stimulus	1	163	0.36	0.54		0.0002
Class:Time	1	163	17.80	4.05e-05	*	0.02
Stimulus:Time	1	163	8.28	0.004	*	0.007
Class:Stimulus:Time	1	163	3.25	0.07		0.003

Supplementary Table 11: Day 1 ANOVA

1) Class fast

Effect	*DFn*	*DFd*	*F*	*p*	*p<.05*	*ges*
Stimulus	1	138	134.66	3.75e-22	*	0.09
Time	1	138	275.29	1.11e-34	*	0.34
Stimulus:Time	1	138	1.80	0.18		0.001

2) Class slow

Effect	*DFn*	*DFd*	*F*	*p*	*p<.05*	*ges*
Stimulus	1	25	29.96	1.09e-05	*	0.14
Time	1	25	13.81	0.001	*	0.08
Stimulus:Time	1	25	6.991045	0.01	*	0.04

Supplementary Table 12: Day 1 Within-Class ANOVA

Effect	*DFn*	*DFd*	*F*	*p*	*p<.05*	*ges*
Class	1	163	88.32	4.92e-17	*	0.26
Stimulus	1	163	109.38	6.53e-20	*	0.06
Time	1	163	166.62	1.03e-26	*	0.14
Class:Stimulus	1	163	4.24	4.08e-02	*	0.002
Class:Time	1	163	4.37	3.80e-02	*	0.004
Stimulus:Time	1	163	21.18	8.35e-06	*	0.008
Class:Stimulus:Time	1	163	9.45	2.46e-03	*	0.004

Supplementary Table 13: Day 2 ANOVA

1) Class fast

Effect	*DFn*	*DFd*	*F*	*p*	*p<.05*	*ges*
Stimulus	1	138	127.41	2.44e-21	*	0.07
Time	1	138	369.77	7.23e-41	*	0.30
Stimulus:Time	1	138	94.63	2.36e-17	*	0.04

2) Class slow

Effect	*DFn*	*DFd*	*F*	*p*	*p<.05*	*ges*
Stimulus	1	25	27.80	1.84e-05	*	0.16
Time	1	25	29.14	1.33e-05	*	0.19
Stimulus:Time	1	25	0.65	0.42		0.001

Supplementary Table 14: Day 2 Within-Class ANOVA

Effect	*DFn*	*DFd*	*F*	*p*	*p<.05*	*ges*
Class	1	163	30.66	1.20e-07	*	0.10
Stimulus	1	163	35.83	1.32e-08	*	0.01
Time	1	163	158.21	8.63e-26	*	0.16
Class:Stimulus	1	163	8.82	0.003	*	0.004
Class:Time	1	163	0.02	0.87		0.00003
Stimulus:Time	1	163	24.42	1.90e-06	*	0.01
Class:Stimulus:Time	1	163	5.62	0.01	*	0.002

Supplementary Table 15: Day 3 ANOVA

1) Class fast

Effect	*DFn*	*DFd*	*F*	*p*	*p<.05*	*ges*
Stimulus	1	138	15.51	1.29e-04	*	0.008
Time	1	138	288.72	1.21e-35	*	0.26
Stimulus:Time	1	138	10.79	0.001	*	0.005

2) Class slow

Effect	*DFn*	*DFd*	*F*	*p*	*p<.05*	*ges*
Stimulus	1	25	17.16	0.0003	*	0.07
Time	1	25	26.15	2.77e-05	*	0.28
Stimulus:Time	1	25	13.68	0.001	*	0.04

Supplementary Table 16: Day 3 Within-Class ANOVA

Predictor	*OR*	*2.5%*	*97.5%*
Intercept	0.171	0.103	0.268
Male	1.595	0.535	4.245

Predictor	*Estimate*	*Std. Error*	*z value*	*p*
Intercept	–1.766	0.242	–7.300	< .001
Male	0.467	0.520	0.898	0.369

Supplementary Table 17: Logistic regression results with class membership as the dependent variable and sex as predictor.

Lonsdorf et al. dataset

Effect	*DFn*	*DFd*	*F*	*p*	*p<.05*	*ges*
Class	1	108	51.06	1.10e-10	*	0.19
Stimulus	1	108	47.68	3.59e-10	*	0.06
Time	1	108	28.004	6.40e-07	*	0.04
Class:Stimulus	1	108	0.38	0.53		0.0005
Class:Time	1	108	6.40	0.01	*	0.01
Stimulus:Time	1	108	8.21	0.005	*	0.008
Class:Stimulus:Time	1	108	6.88	0.009	*	0.007

Supplementary Table 18: Day 1 ANOVA

1) Class fast

Effect	*DFn*	*DFd*	*F*	*p*	*p<.05*	*ges*
Stimulus	1	86	57.23	3.96e-11	*	0.08
Time	1	86	75.41	2.16e-13	*	0.15
Stimulus:Time	1	86	0.09	0.75		0.0001

2) Class slow

Effect	*DFn*	*DFd*	*F*	*p*	*p<.05*	*ges*
Stimulus	1	22	10.64	0.003	*	0.09
Time	1	22	2.15	0.15		0.01
Stimulus:Time	1	22	4.45	0.046	*	0.04

Supplementary Table 19: Day 1 Within-Class ANOVA

Effect	*DFn*	*DFd*	*F*	*p*	*p<.05*	*ges*
Class	1	108	25.54	1.77e-06	*	0.09
Stimulus	1	108	8.63	0.004	*	0.009
Time	1	108	95.69	1.45e-16	*	0.22
Class:Stimulus	1	108	0.46	0.49		0.0005
Class:Time	1	108	2.39	0.12		0.007
Stimulus:Time	1	108	0.01	0.90		0.00001
Class:Stimulus:Time	1	108	3.17	0.07		0.003

Supplementary Table 20: Day 2 ANOVA

1) Class fast

Effect	*DFn*	*DFd*	*F*	*p*	*p<.05*	*ges*
Stimulus	1	86	7.10	9.16e-03	*	0.008
Time	1	86	149.89	1.53e-20	*	0.37
Stimulus:Time	1	86	4.81	0.03	*	0.006

2) Class slow

Effect	*DFn*	*DFd*	*F*	*p*	*p<.05*	*ges*
Stimulus	1	22	2.64	0.11		0.01
Time	1	22	23.65	7.33e-05	*	0.20
Stimulus:Time	1	22	0.61	0.44		0.004

Supplementary Table 21: Day 2 Within-Class ANOVA

Effect	*DFn*	*DFd*	*F*	*p*	*p<.05*	*ges*
Class	1	108	17.76	5.19e-05	*	0.06
Stimulus	1	108	3.67	5.78e-02		0.005
Time	1	108	59.59	6.26e-12	*	0.15
Class:Stimulus	1	108	0.30	0.58		0.0004
Class:Time	1	108	1.25	0.26		0.003
Stimulus:Time	1	108	1.85	0.17		0.001
Class:Stimulus:Time	1	108	0.05	0.81		0.00005

Supplementary Table 22: Day 3 ANOVA

1) Class fast

Effect	*DFn*	*DFd*	*F*	*p*	*p<.05*	*ges*
Stimulus	1	86	9.09	0.003	*	0.01
Time	1	86	92.05	3.03e-15	*	0.27
Stimulus:Time	1	86	1.76	0.18		0.002

2) Class slow

Effect	*DFn*	*DFd*	*F*	*p*	*p<.05*	*ges*
Stimulus	1	22	0.33	0.57		0.003
Time	1	22	14.61	0.0009	*	0.16
Stimulus:Time	1	22	0.52	0.47		0.003

Supplementary Table 23: Day 3 Within-Class ANOVA

Predictor	*OR*	*2.5%*	*97.5%*
Intercept	0.220	0.116	0.388
Male	1.621	0.623	4.144

Predictor	*Estimate*	*Std. Error*	*z value*	*p*
Intercept	–1.513	0.306	–4.937	< .001
Male	0.483	0.479	1.008	0.313

Supplementary Table 24: Logistic regression results with class membership as the dependent variable and sex as predictor.

[x,y,z]	Z	k_E	Regions
**-52 14 0**	5.23	21	Left inferior frontal gyrus (IFG)
**-14 10 -8**	5.21	24	Left ventral striatum
**-36 -2 38**	4.84	46	Left precentral gyrus
**-46 24 24**	4.79	33	Left middle frontal gyrus (MFG)
**36 30 12**	4.71	10	Right inferior frontal gyrus (IFG)
**-34 22 14**	4.49	15	Left inferior frontal gyrus (IFG)
**-54 -38 46**	4.37	108	Left supramarginal gyrus (SMG)
**40 -32 12**	4.34	45	Right planum temporale
**-58 12 20**	4.23	22	Opercular part of left inferior frontal gyrus (IFG)
**44 -66 -30**	4.19	16	Right cerebellum exterior
**28 10 56**	4.01	15	Right middle frontal gyrus (MFG)
**-32 28 -10**	3.90	12	Left orbital frontal cortex
**-38 36 8**	3.82	15	Left inferior frontal gyrus (IFG)
**-6 22 38**	3.78	10	Left supplementary motor cortex (SMA)
**-8 6 68**	3.65	12	Left superior frontal gyrus (SFG)
**-42 -34 56**	4.32	21	Left postcentral gyrus
**-2 28 -8**	4.19	13	Left anterior cingulate gyrus (ACC)

Supplementary Table 25: Exploratory whole-brain analysis (CS offset by time by class).
Voxelwise p<0.001 uncorrected, minimum cluster size k≥10; peak-level.

While our analyses of CS offset-related activations suggest that dopaminergic PE signaling in the ventral striatum is attenuated or delayed in the slow extinguishers, possibly requiring a higher threshold of expectancy violation or a longer accumulation of evidence before a reliable EPE is generated, it is also conceivable that the late-emerging ventral striatal signal in slow extinguishers could reflect a compensatory or downstream process rather than a canonical EPE. For example, other brain regions such as the ventromedial prefrontal cortex (vmPFC) might initially detect the absence of threat through top-down mechanisms, subsequently activating the VS indirectly once safety information is established (1,2). For this reason, we also examined the reciprocal connectivity (vmPFC to VS peak). Across participants, vmPFC to VS peak connectivity increased over time, with significant main effects in the mid and late windows (within VS peak at mid: Z=3.79, p_{SVC}=.004; late: Z=3.52, p_{SVC}=.012 after small-volume correction for multiple comparisons).

Whole-brain PPIs using the vmPFC as the seed revealed robust connectivity during early and mid extinction including the broader striatum, bilateral anterior insula, supramarginal gyrus, ACC/dmPFC, and bilateral precuneus, whereas the pattern decreased by late extinction (Supplementary Table 26 for p_{FWE} corrected clusters).

When comparing fast and slow extinguishers, a class-dependent pattern emerged (Supplementary Fig. 3). Fast extinguishers specifically showed stronger vmPFC to VS peak coupling early in extinction, whereas slow extinguishers showed stronger coupling late in extinction (class by time interaction: $F_{2,240}$=7.49, p<.001, generalized η^2=.027; class: $F_{1,120}$=0.11, p=.74, generalized η^2<.001; time: $F_{2,240}$=5.94, p=.003, generalized η^2=.022, n=122). This pattern is not in agreement with the above idea that the vmPFC is involved in detecting safety specifically in slow extinguishers. A likely interpretation of the time-dependent class differences in vmPFC to VS peak coupling could be methodological, namely that coupling is most easily detected during the time frame of the extinction phase where the VS peak signal is strongest (early in fast extinguishers, late in slow extinguishers).

To test whether vmPFC–VS connectivity predicts extinction success, we derived differential SCRs at the end of extinction (extinction success) for the MRI subsample (n=122). For the vmPFC-VS peak, early connectivity (first time bin) was computed and entered into a linear regression predicting extinction success. Early vmPFC–VS connectivity did not significantly predict extinction success (β = −0.039, SE = 0.026), t(120) = −1.52, p = .131, accounting for little variance in the outcome (R^2 = .019, adjusted R^2 = .011).

vmPFC seed - VS peak PPI

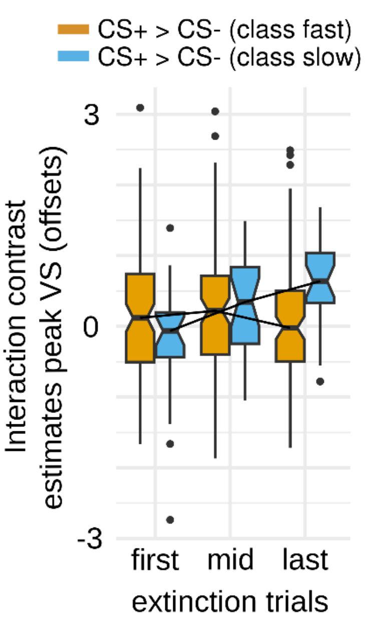

Supplementary Figure 3: Interaction contrast estimates extracted from the target in a reciprocal PPI (vmPFC to VS peak) shows a class specific temporal profile.

Time	x,y,z	Z	k_E	Regions
**Early extinction (first 5 trials)**	32 20 -14	7.37	4598	Right anterior insula, right inferior frontal gyrus (IFG)
	64 -42 26	6.04	1735	Right supramarginal gyrus (SFG)
	4 42 36	5.87	1982	Medial superior frontal gyrus (SFG), anterior cingulate gyrus (ACC)
	54 -22 -8	5.76	349	Right middle temporal gyrus (MTG)
	-12 -78 -28	5.61	611	Left cerebellum exterior
	-30 14 -12	5.55	1294	Left anterior insula, left putamen
	4 -28 30	5.06	297	Right posterior cingulate gyrus, right middle cingulate gyrus
	12 -64 34	4.92	121	Right precuneus
	-64 -36 26	4.68	292	Left supramarginal gyrus (SMG)
**Mid extinction (middle 5 trials)**	8 46 18	6.01	3011	Medial superior frontal gyrus (SFG), anterior cingulate gyrus (ACC)
	28 22 -10	5.70	2014	Right anterior insula, right putamen
	-26 12 2	5.26	1170	Left putamen
	64 -22 -10	5.16	1122	Right middle temporal gyrus (MTG), right angular gyrus
	-12 38 10	4.79	121	Left anterior cingulate gyrus
	-30 44 36	4.37	247	Left middle front gyrus (MFG), left superior frontal gyrus (SFG)
	-16 -82 -28	4.36	302	Left cerebellum exterior
**Late extinction (last 5 trials)**	44 22 -8	5.32	752	Right inferior frontal gyrus (IFG), right anterior insula, right frontal operculum

	-10 10 14	4.67	225	Left caudate
	-28 22 -4	4.48	99	Left anterior insula
	2 32 46	4.47	166	Middle superior frontal gyrus (SFG), right supplementary motor cortex (SMA)
	-40 16 0	4.43	69	Left frontal operculum
	38 48 20	4.42	342	Right middle frontal gyrus (MFG)
	54 -46 50	4.33	328	Right supramarginal gyrus (SMG), right angular gyrus
	44 8 54	4.08	85	Right middle frontal gyrus (MFG)
	14 14 12	4.01	125	Right caudate

Supplementary Table 26: Significant whole-brain PPI clusters for the main effect of connectivity (seeded in the vmPFC) at early, mid, and late extinction. Analyses were thresholded at $p < .001$ (uncorrected) at the voxel level with cluster-level FWE correction ($p_{FWE} < .05$). For each significant cluster, the table lists its anatomical label, peak Z value, cluster extent (kE), and MNI peak coordinates (x,y,z). All reported clusters survive FWE correction at the cluster level. Coordinates reflect the voxel of maximum statistical significance within each cluster.

References

1. Andres E, Chuan-Peng H, Gerlicher AMV, Meyer B, Tüscher O, Kalisch R. Replication study on the role of dopamine-dependent prefrontal reactivations in human extinction memory retrieval. Nat Commun. 2024 Mar 27;15(1):2699.

2. Gerlicher AMV, Tüscher O, Kalisch R. Dopamine-dependent prefrontal reactivations explain long-term benefit of fear extinction. Nat Commun. 2018 Dec;9(1):4294–4294.
